# *K* Nearest Neighbor Algorithm Coupled with Metabonomics to Study the Therapeutic Mechanism of Sendeng-4 in Adjuvant-Induced Rheumatoid Arthritis Rat

**DOI:** 10.1155/2018/2484912

**Published:** 2018-02-22

**Authors:** Xiye Wang, Dan Li, Mingyang Jiang, Zhili Pei, Liang Xu

**Affiliations:** ^1^Inner Mongolia Key Laboratory for the Natural Products Chemistry and Functional Molecular Synthesis, College of Chemistry and Chemical Engineering, Inner Mongolia University for the Nationalities, Tongliao, Inner Mongolia, China; ^2^College of Computer Science and Technology, Inner Mongolia University for the Nationalities, Tongliao, Inner Mongolia, China

## Abstract

As a traditional Mongolian medicine, Sendeng-4 (SD) has been widely used to treat rheumatoid arthritis (RA) in Inner Mongolia and exhibits a good curative effect. Unfortunately, due to geographical factors, it is difficult to popularize this drug throughout the whole country, and the mechanism of action of SD has been unclear. In this study, a serum metabolite profile analysis was performed to identify potential biomarkers associated with adjuvant-induced RA and investigate the mechanism of action of SD. Ultraperformance liquid chromatography coupled with quadrupole time-of-flight mass spectrometry (UPLC-Q-TOF-MS) was performed for the metabonomics analysis. *K* nearest neighbor (KNN) models were established in both positive and negative spectra for classifying data from the control, model, and SD administration groups. Accuracy rate for classification was 95.8% in positive ion mode and 91.7% in negative ion mode. Orthogonal partial least squares discriminant analysis (OPLS-DA) enabled the identification of 12 metabolites as potential biomarkers of adjuvant-induced RA. After treatment with SD, the levels of uridine triphosphate, calcitroic acid, dynorphin B (6-9), and docosahexaenoic acid were restored to normal, indicating that SD likely ameliorated RA by regulating the levels of these biomarkers. This study identified early biomarkers of RA and elucidated the underlying mechanism of action of SD, which is worth further investigation for development as a clinical therapy.

## 1. Introduction

Rheumatoid arthritis (RA) is an autoimmune inflammatory disease. The main clinical manifestations are chronic, symmetrical, and synovial arthritis and extra-articular disease. RA, a progressive disease, occurs in small joints such as the hand, wrist, and foot and causes joint deformities and loss of joint function [1]. Currently, the treatment of RA mainly relies on Western medicine such as the nonsteroidal drug, diclofenac [2]; antirheumatic drug, methotrexate [3]; and glucocorticoid drug, dexamethasone [4]. These Western medicines are highly efficacious but are accompanied by toxic side effects. In addition, Western medicine can only temporarily relieve or eliminate the pain and cannot cure RA fundamentally.

Mongolian medicine has its own unique theory and method in the treatment of RA and can regulate the physiological activity of the human body from the perspective of allometric function. Mongolian medicine Sendeng-4 (SD) is comprised of* Xanthoceras sorbifolia*,* Toosendan fructus*,* Gardeniae fructus*, and* Chebulae fructus* at a ratio of 5 : 3 : 1 : 1. SD is mainly used in the treatment of gout, rheumatism, joint grasserie, and edema [5, 6]. Although SD has a good curative effect on RA which is not prone to relapse after recovery, the popularization of SD is challenging due to geographical factors and incomplete understanding about its mechanism of action.

Metabonomics is the science about the changing roles of endogenous metabolic substances in organisms and has been widely used in the study of the mechanism of action of drugs [7–9]. As a burgeoning metabonomics approach, the *K* nearest neighbor (KNN) classification algorithm is one of the simplest methods in data mining classification. The nearest neighbor of *K* means that each sample can be represented by its nearest *K* samples. The classification model of KNN is simple and effective. The principle of KNN describes that if there is a test data to be classified, the most similar known data is found by comparison to the classifying data, and the category of the data to be classified is judged based on the type of known data [10, 11]. The traditional metabonomics method is principal component analysis (PCA). PCA loses a considerable amount of raw data—the cumulative contribution rate of the first few principal components must be high or else the model is not qualified. KNN does not lose any raw data, which gives it a stronger advantage over traditional statistical methods in the classification of multidimensional data. At present, KNN classification has been widely used in molecular biology research of RA. Inhibition of TNF-alpha converting enzyme (TACE) is one of the most direct and effective therapies for RA. Therefore, the screening of TACE inhibitors has become a very important task. KNN was used to classify TACE inhibitors and noninhibitors, and the KNN model gave a classification accuracy of 98.32% [12]. Peripheral blood gene expression profiles (PBGE) were used to predict disease severity in early RA patients, based on KNN classification, and results showed that KNN effectively predicted RA severity [13]. The bone lesion volume (BeltaBLV) in RA patients' hands would be used to evaluate RA progression. This study demonstrated that the combination of multispectral (MS) MRI analysis method and KNN classification provided the quantitative tool for the BeltaBLV [14]. So far, work regarding RA metabonomics coupled with KNN classification has not been reported. In this study, KNN algorithm combined with the metabonomics method was applied to classify data from different groups. Potential biomarkers were identified using the OPLS-DA model. Our study revealed the mechanism of SD in RA treatment by studying the metabolic pathway of potential biomarkers.

## 2. Materials and Methods

### 2.1. Chemicals and Reagents

SD was provided by the Mongolian Medicine Manufacturing Room of the Affiliated Hospital of Mongolia University for the Nationalities (Tongliao, China). SD powder was dissolved in 0.5% carboxymethyl cellulose sodium (CMC) aqueous solution up to concentrations of 0.43 g/mL and stored at 4°C for animal experimentation. Complete Freund's adjuvant (CFA) was purchased from Sigma Chemical Co. (St. Louis, MO, USA). Methanol and formic acid (Fisher Scientific, UK) were HPLC-grade. The kits for superoxide dismutase (SOD, H001-3-201705-96), hydroxyl radical (OH^•^, H081-201705-96), tumor necrosis factor-*α* (TNF-*α*, H052-201704-96), and interleukin-6 (IL-6, H007-201706-96) assays were purchased from Nanjing Jiancheng Bioengineering Institute (Nanjing, China).

### 2.2. Adjuvant-Induced Arthritis Model Establishment and Treatment

The study was approved by the ethics committee of the Affiliated Hospital of Inner Mongolia University for the Nationalities (NMMZDX2017[K]0013). Male Wistar rats (200 ± 10 g) were provided by YiSi Laboratory Animal Technology Co., Ltd., (Changchun, China). All animals were reared under standard conditions (21 ± 2°C, daily sunshine for 14 hours) with free access to rodent chow and water in the Affiliated Hospital and allowed to acclimatize in metabolism cages for 1 week prior to experiment. The rats were divided into three groups: control, model, and SD administration groups (CG, MG, and SG, resp.), with eight rats in each group. Prior to the experimentation, all the rats were acclimated for 7 days. On Day 1, the rats in MG and SG were intradermally injected with 0.1 mL CFA in the right posterior toe and the rats in CG were injected with 0.1 mL saline. After 7 days, the rats in MG and SG were injected with 0.1 mL CFA again. On Day 14, the rats in SG were administered SD at doses of 0.43 g/kg/day for 35 consecutive days, and on Day 49, all the rats were euthanized. Blood was collected from the hepatic portal vein and centrifuged at 3500 rpm for 10 min at 4°C. The supernatants were immediately frozen, stored at −20°C, and thawed before analysis. Arthrodial cartilage was fixed in 10% formaldehyde for paraffin-embedding.

### 2.3. Biochemical and Histological Analysis

Arthrodial cartilage was cut and processed for hematoxylin and eosin (H&E) staining. H&E staining was performed to examine the pathological changes of arthrodial cartilage, and the specimens were observed by microscopy. The levels of SOD, OH^•^, TNF-*α*, and IL-6 were measured using a Multiskan FC Microplate Reader (Fisher Scientific, UK).

### 2.4. Serum Sample Preparation

The serum samples were thawed before analysis, and 100-*μ*L aliquots were added to 400 *μ*L acetonitrile, followed by vortexing for 30 s and centrifugation at 12000 rpm for 10 min at 4°C. The supernatant was subsequently filtered through a 0.22-*μ*m filter membrane.

### 2.5. UPLC-MS Conditions

A Waters Acquity UPLC system coupled with a Q-TOF Xevo G2-S high definition mass spectrometer (Waters, UK) was used for the metabonomics analysis. The Waters Acquity UPLC BEH C_18_ Column (1.7-*μ*m, 2.1 mm × 50 mm, Waters, USA) was maintained at 40°C with a flow rate of 0.4 mL·min^−1^ for the separation. The mobile phases were 0.1% formic acid in deionized water (A) and methanol (B). The gradient elution with B was performed according to the following schedule: 8–80% B for 0–3 min, 80–100% B for 3–6 min, 100% B for 6–8 min, and 100–8% B for 8-9 min, followed by maintaining at 8% B for 2 min. The sample injection volume was 10 *μ*L.

For the UPLC-high-definition MS (HDMS) analysis, the optimal conditions were the positive ion mode with source and desolvation gas temperatures of 100°C and 400°C, respectively. Nitrogen was used as the cone and desolvation gas at flow rates of 50 and 800 L/h, respectively. The capillary, cone, and offset voltages were set at 3.0 kV, 40 V, and 80 V, respectively. In the negative ion mode, the source and desolvation gas temperatures were 80°C and 150°C. Nitrogen was the cone and desolvation gas at flow rates of 0 L/h and 600 L/h, respectively. The capillary, cone, and offset voltages were set at 2.5 kV, 40 V, and 80 V, respectively.

The MS data were collected in the full-scan mode in the mass range of 100–1000 Da and at a scan time of 0.5 s. The MS^2^ collision energy was set at 15–35 eV, and a lock-mass of leucine enkephalin at a concentration of 200 pg/mL in acetonitrile : water (50 : 50, v/v) was used for the positive ([M + H]^+^ = 556.2771) and negative ([M − H]^−^ = 554.2615) ion modes with a lock spray interface. The data were collected in the continuum mode, and the lock spray frequency was set at 10 s.

### 2.6. Data Analysis

A pooled quality control (QC) sample was prepared by mixing aliquots (20 *μ*L) of each sample to monitor instrument stability. Every day, after the instrument was calibrated, five QC samples were analyzed to test the stability of the instrument. The MassLynx V4.1 software was used for peak detection and alignment. After recognition and alignment, all the data acquired were normalized to the total ion intensity of each chromatograph.

The data matrix was established by aligning the peaks with the exact mass/retention time pair from each data file in the data set with their associated normalized peak areas. Then, the data matrix was analyzed using pattern recognition. Matlab 2012 software was applied for classification of data based on the KNN algorithm. EZinfo 2.0 software was used for OPLS-DA while an independent sample *t*-test was performed using the statistical package for the social sciences (SPSS) version 17.0. HemI software was used to analyze the heatmap of the metabolites [15]. The Human Metabolome Database (HMDB), METLIN, and Kyoto Encyclopedia of Genes and Genomes (KEGG) metabonomics databases were used to identify potential biomarkers. The tandem MS (MS/MS) spectra were compared with MS/MS information from these databases to verify the structure of the putative metabolites.

## 3. Results and Discussion

### 3.1. Biochemistry and Histology

**Figure 1:**
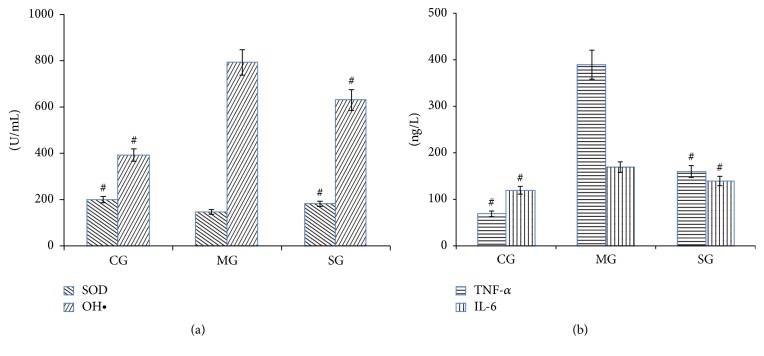
Indices of oxidant stress (a) and cytokine levels (b) in rat serum. Data are shown as mean ± standard deviation (*n* = 8). CG: control group; MG: model group; SG: SD administration group. ^#^*p* < 0.05 compared with MG.

Figure 1 shows the serum biochemical parameters derived from each group. SOD is an important antioxidant enzyme that can scavenge free radicals in organisms. As indicated in Figure 1(a), the content of SOD decreased and the content of OH^•^ increased in MG compared to those in CG, meaning that the antioxidant system in RA rats was compromised. After SD administration, the contents of SOD and OH^•^ were restored, indicating effective antioxidant activity for SD.

Inflammatory mediators TNF-*α* and IL-6 play an important role in the pathogenesis of RA. They are produced by infiltration of immune cells and excessive secretion of matrix metalloproteinases (MMPs). These mediators have a strong erosive effect on cartilage and bone joints [16]. As shown in Figure 1(b), the contents of TNF-*α* and IL-6 increased significantly in MG compared to those in CG. However, in SG, the contents of TNF-*α* and IL-6 decreased compared to those in MG, indicating that SD has an immunosuppressive effect.

**Figure 2:**
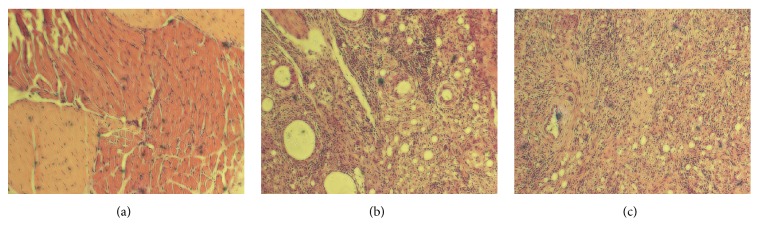
Histopathological change in rat arthrodial cartilage. Photomicrographs show representative cartilage sections stained with hematoxylin and eosin (H&E, 200x). (a) Control group; (b) model group; (c) SD administration group.

Figure 2(a) shows the arthrodial cartilage in CG and a large number of pannus (yellow circle in Figure 2(b)) can be seen in MG. Pannus is formed by plasma cells, macrophages, and lymphocytes and can release immunoglobulins and rheumatoid factor (RF). Pannus not only hinders bone's ability to obtain nourishment through the synovial membrane, but also releases a variety of inflammatory mediators and proteolytic enzymes to erode the articular cartilage, subchondral bone, ligament, and tendon tissue, causing destruction of articular cartilage, subchondral osteolysis, joint capsule relaxation damage, joint dislocation, joint fusion, and ossification [17]. After administration of SD (Figure 2(c)), the number and volume of panni decreased in SG, indicating that SD alleviated the symptoms of RA in rats.

### 3.2. Application of KNN Algorithm for Classification

**Figure 3:**
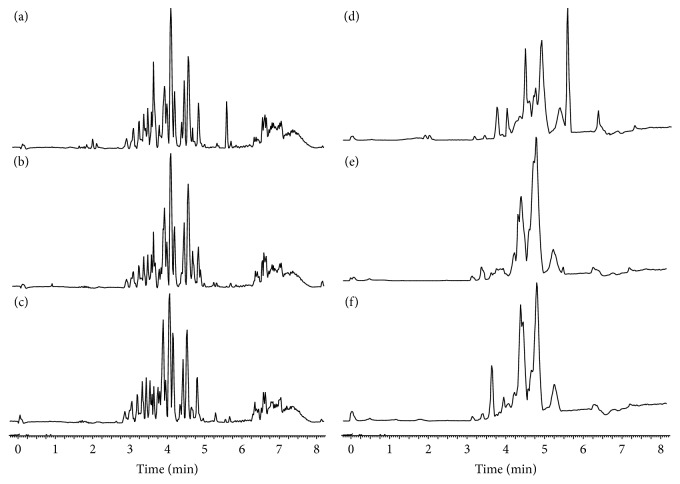
Serum base peak chromatograms of various groups. (a) Control group (CG), (b) model group (MG), and (c) SD administration group (SG) in positive mode and (d) CG, (e) MG, and (f) SG in negative mode.

The metabolite characteristics were investigated using positive and negative ion modes. The serum base peak intensity (BPI) chromatograms of the CG, MG, and SG in the positive and negative ion modes are described in Figure 3. Despite the obvious differences in the chromatogram, the multivariate analysis distinguished the three groups more accurately than other analyses.

**Figure 4:**
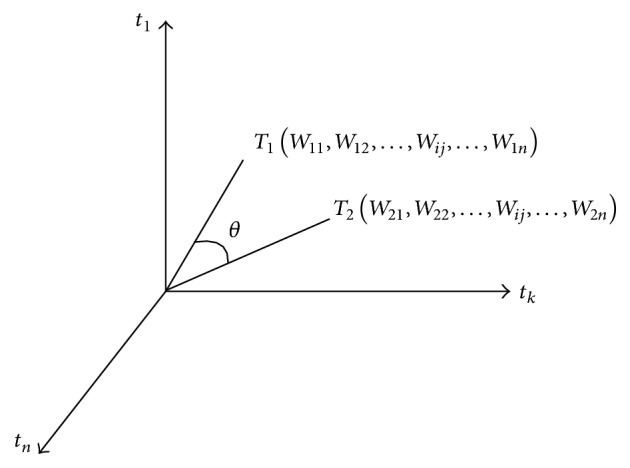
KNN algorithm model in this experiment, where *T*_1_(*W*_11_, *W*_12_,…, *W*_*ij*_,…, *W*_1*n*_) and *T*_2_(*W*_21_, *W*_22_,…, *W*_*ij*_,…, *W*_2*n*_) represent two different omics data, *W*_*ij*_ represents the weight value of the *j*th feature of the *i*th omics data, and *θ* represents the angle of the two omics data vectors (*i* can be seen as one sample, and *j* can be seen as one biomarker in this sample).

In this experiment, the KNN algorithm was used for classification of metabonomics data. Each group of data in the vector space model was expressed as a vector form. As shown in Figure 4, the calculation of the similarity (distance) of the two sets of data was converted into the calculation of the vector.

Methods for calculating similarity are Euclidean distance, Vector Inner Product, and Included Angle Cosine, and their formulas are as follows:

*Euclidean Distance*

(1) $$\begin{array}{c} Sim\left(\;d_{1},d_{2}\;\right)=\sqrt{\overset{n}{\underset{i=1}{\sum }}{\left(W_{1i}-W_{2i}\right)}^{2}}, \end{array}$$

where *W*_1*i*_ and *W*_2*i*_ are weights representing the corresponding feature in the omics data vectors *d*_1_ and *d*_2_. If the calculated value is smaller, the distance between the two omics data vectors is smaller, and the similarity of the two omics data is greater.

*Vector Inner Product*

(2) $$\begin{array}{c} Sim\left(\;d_{1},d_{2}\;\right)=\overset{n}{\underset{i=1}{\sum }}\left(\;W_{1i}*W_{2i}\;\right), \end{array}$$

where *W*_1*i*_ and *W*_2*i*_ are weights representing the corresponding feature in the omics data vectors *d*_1_ and *d*_2_. The inner product represents the projection of a vector on another vector. When the inner product is used as the similarity formula and the inner product is larger, the similarity of the two omics data is greater.

*Included Angle Cosine*

(3) $$\begin{array}{c} Sim\left(\;d_{1},d_{2}\;\right)=\frac{\sum_{i=1}^{n}\left(W_{1i}*W_{2i}\right)}{\sqrt{\sum_{i=1}^{n}\left(W_{1i}^{2}\right)*\sum_{i=1}^{n}\left(W_{2i}^{2}\right)}}, \end{array}$$

where *W*_1*i*_ and *W*_2*i*_ are weights representing the corresponding feature in the omics data vectors *d*_1_ and *d*_2_. If the angle is smaller between the two vectors and the cosine value is larger, it is likely that the two groups of data belong to the same category. On the contrary, if the cosine value is smaller, the two groups of data are not likely to belong to the same category. In this paper, we use the angle cosine algorithm to calculate the similarity between two sets of metabonomics data.

In the KNN-based metabolomics data classification study, the cross-validation method is applied to complete the KNN model classification evaluation. Part of the sample is used as training set data, and the rest is used as testing data to calculate classification accuracy rate. The above process needs to be performed until every sample is predicted once and only once. The purpose of cross-validation is to obtain a reliable and stable model. The method of 4-fold cross-validation was used in this study. The group data set was divided into four sets, and the verification experiments were completed four times. One copy of the data was used to test the model in each experiment, while the remaining three data copies were used to train the model.

Ultimately, two KNN models were established by Matlab 2012 software (one model for positive ion mode, the other for negative ion mode). The data matrix contained 3253 dimensional data points in positive ion mode and 1045 dimensional data points in negative ion mode. Twenty-four samples were assigned to three categories. The classification accuracy rates were 95.8% (23/24) in positive ion mode (eight correct samples in CG, seven correct samples in MG, eight correct samples and one incorrect MG sample in SG) and 91.7% (22/24) in negative ion mode (eight correct samples in CG, six correct samples in MG, eight correct samples and two incorrect MG samples in SG). These results show that the two KNN models are accurate and reliable.

**Figure 5:**
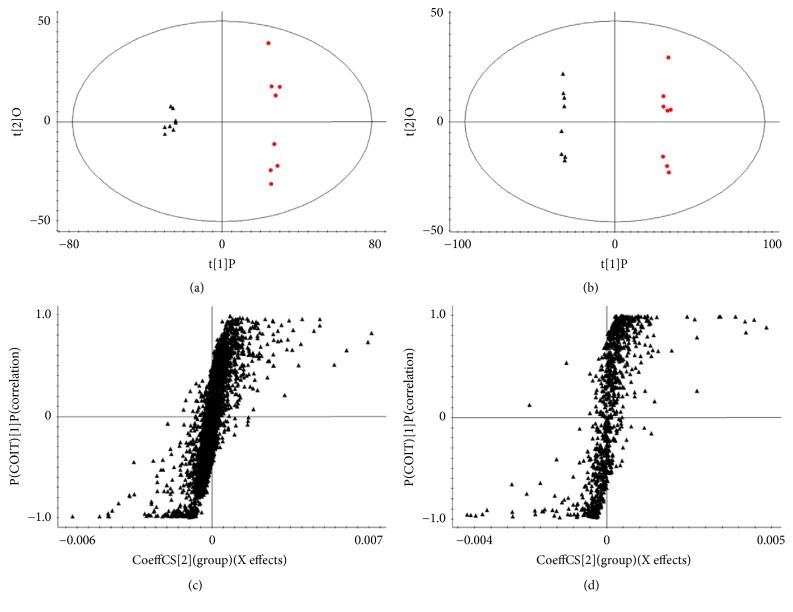
Orthogonal partial least squares discriminant analysis (OPLS-DA) score plots of serum metabolic profiling of all groups. (black triangle) CG and (red circle) MG in (a) positive mode and (b) negative mode. OPLS-DA S-plots in (c) positive and (d) negative modes. CG: control group; MG: model group (*n* = 8 in each group).

An OLPS-DA model was used to detect potential biomarkers and investigate the effect of SD on the metabolic pathway in RA rats. The OPLS-DA score plots are shown in Figures 5(a) and 5(b). As indicated by *R*^2^*Y* = 0.9974 and *Q*^2^ = 0.9896 in the positive ion mode and *R*^2^*Y* = 0.9918 and *Q*^2^ = 0.9775 in the negative ion mode, the model was both reliable and predictive.

### 3.3. Identification of Potential Biomarkers

**Figure 6:**
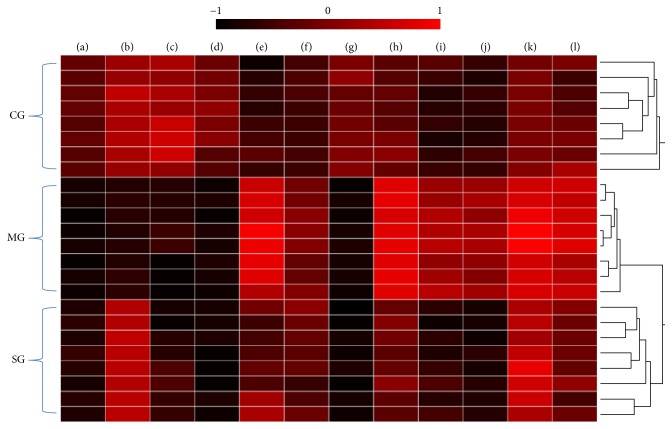
Heatmap of potential biomarkers in all groups. CG: control group; MG: model group; SG: SD administration group. (a) Guanosine pentaphosphate adenosine; (b) dynorphin B (6-9); (c) bilirubin glucuronide; (d) LysoPE (0:0/24:0); (e) guanosine diphosphate; (f) stearic acid; (g) alpha-tocotrienol; (h) docosahexaenoic acid; (i) calcitroic acid; (j) uridine triphosphate; (k) MG (0:0/14:0/0:0); (l) eicosenoic acid.

The S-plots of the OPLS-DA are shown in Figures 5(c) and 5(d), and the variables contributed highly to the differentiation situated at the edges of the plots. The spots, which showed VIP values > 1.0 and *p* < 0.05, were considered to represent potential biomarkers. We identified 12 metabolites that showed significant differences between the CG and MG as potential biomarkers. These metabolites consisted of four and eight points selected based on the data of the positive and negative mode analyses, respectively. Among these metabolites, the levels of docosahexaenoic acid, stearic acid, eicosenoic acid, MG (0:0/14:0/0:0), calcitroic acid, uridine triphosphate, and guanosine diphosphate in the MG were higher than the levels in the CG. In addition, the levels of dynorphin B (6-9), guanosine pentaphosphate adenosine, bilirubin glucuronide, LysoPE (0:0/24:0), and alpha-tocotrienol in the MG were lower than those in the CG. However, after treatment with SD, uridine triphosphate, calcitroic acid, dynorphin B (6-9), and docosahexaenoic acid returned to normal levels. This observation indicates that SD may prevent the pathological process of RA rats by regulating the disturbed metabolic pathway of these four potential biomarkers. The heatmap of the potential biomarkers is described in Figure 6. All the biomarker information is summarized in Table 1.

### 3.4. Biological Relevance

Uridine triphosphate (UTP) is the raw material for RNA synthesis. The combination of UTP and vitamin B12 has been reported to have a significant effect on the treatment of compressive neuralgias. Compared with vitamin B12 administration alone, the combination of UTP and vitamin B12 had more statistically significant advantages in alleviating pain in patients with no serious adverse events during the study period [18]. Dysregulation of adipogenesis of bone marrow-derived stromal cells (BMSCs) and osteogenesis can lead to osteoporosis. After activating the P2Y2 receptor, UTP can retard the progression of osteoporosis through regulating the osteogenic and adipogenic differentiation of BMSCs [19]. Therefore, the dysregulation of UTP in the MG is likely to result in osteoarthropathy.

Calcitroic acid is the metabolite of 1,25(OH)_2_D_3_, which is the active form of vitamin D. 1,25(OH)_2_D_3_ has many physiological functions such as increasing the absorption of calcium and phosphorus, promoting growth and bone calcification, maintaining normal levels of citrate in blood, and preventing the loss of amino acid in renal metabolism [20]. The immune response to antigens derived from* Aspergillus fumigatus* can cause the allergic disease, allergic bronchopulmonary aspergillosis (ABPA), which is accompanied by an increased interleukin-13 response in blood CD4+ T cells. 1,25(OH)_2_D_3_ can inhibit this allergic response and improve the ABPA patient's condition [21]. In addition, 1,25(OH)_2_D_3_ has immunomodulatory and anti-inflammatory activity. Research shows that 1,25(OH)_2_D_3_ plays a crucial role in the progression of many autoimmune diseases such as rheumatoid arthritis, multiple sclerosis (MS), and osteoporosis [22]. Numerous studies have shown that the lack of sunlight and 1,25(OH)_2_D_3_ will lead to MS; thus, 1,25(OH)_2_D_3_ supplementation is a very effective treatment for MS patients [23]. Osteoporosis is highly correlated with atherosclerosis. The parallel progression of the two diseases increases coronary and fracture risks. 1,25(OH)_2_D_3_ deficiency can greatly increase the risk of fracture and result in secondary hyperparathyroidism and coronary artery calcification [24]. The content of calcitroic acid increased significantly in the MG, indicating that the content of 1,25(OH)_2_D_3_ decreased, which reduces the regulatory ability of the immune system.

Dynorphin B (DYN) is an endogenous opioid peptide (EOP) widely distributed in the central nervous system and peripheral nervous system. EOPs are selective for different receptors, and DYN is a type *κ* receptor ligand. Peripheral inflammation of RA causes an increase in the number of immune cells (T cells, B lymphocytes, macrophages, monocytes). The immune cells that migrate to the inflammatory region can synthesize large amounts of EOP. Meanwhile, inflammatory responses promote the synthesis of EOP receptors in the dorsal root ganglion (DRG) and transport these receptors to the peripheral nerve tip of the inflammatory region. After EOP activates the receptors, they can act as the analgesic for RA [25, 26]. Studies have shown that in synovial tissue and immune cells of RA rats, EOPs were released in large quantities, and the expression of their receptors increased in peripheral nerve tips. EOPs and their receptors also participate in the regulation of chronic inflammation [27]. Studies have found that injecting EOP receptor agonists in the knee reduces pain in patients with joint pain, while injecting EOP receptor antagonists in the knee after surgery increases the pain of the joints [28, 29]. EOP may act on the EOP receptors in immune cells and regulate the function of immune cells, which eventually participate in the regulation of inflammatory factors. Levels of EOP are associated with the concentrations and mRNA expression of inflammatory factors such as interleukin-1*β* (IL-1*β*), interleukin-6 (IL-6), and tumor necrosis factor *α* (TNF-*α*). These inflammatory factors can cause pain in the joints, fever, and edema. By utilizing the negative feedback regulation of inflammatory factors and decreasing the excitability of the sensory nerve, EOP regulates inflammatory pain [30]. The content of DYN significantly decreased in the MG, suggesting that it was complexed with the EOP receptor to inhibit inflammation caused by RA.

Docosahexaenoic acid (DHA) is a necessary polyunsaturated fatty acid for the human body. DHA has a variety of biological activities such as assisting brain cell development, slowing aging, improving blood circulation, and reducing blood lipids. In addition, DHA may protect against RA. It was reported that DHA could inhibit the proliferation and differentiation of bone marrow-derived macrophages (BMMs) and induce the apoptosis of mature osteoclasts. Eventually, DHA led to a reduction in the number of bone-resorptive cells [31]. DHA can generate a new type of bioactive lipid mediator through biological derivatization. These endogenous mediators can act on specific G protein-coupled receptors (GPCRs) to inhibit inflammation [32]. The content of DHA significantly increased in the MG, indicating that the body showed a stress response to the inflammation caused by RA.

Dysregulation of UTP, calcitroic acid, DYN, and DHA levels could lead to a series of changes in related metabolic pathways in the body. However, after administration of SD, their levels were close to normal, indicating that SD successfully ameliorated RA by regulating the levels of these four potential biomarkers.

## 4. Conclusions

In summary, SD showed a good therapeutic effect on RA rats induced by adjuvant. The KNN algorithm was used to classify metabonomics data and achieved a high classification accuracy in both positive and negative spectra. The results illustrated that the KNN model in this study was reliable. In total, 12 potential biomarkers were identified, of which UTP, calcitroic acid, DYN, and DHA were considered correlated with the therapeutic effect of SD in RA rats. Further investigation is required to determine the relationship between these four biomarkers and to infer the complete metabolic pathways of SD for RA treatment.

## Figures and Tables

**Table 1:** Potential biomarkers related to RA in rats between CG and MG.

No.	RT (min)	Measured mass	Error (ppm)	Molecular formula	Identified	VIP	ESI mode
(1)	3.47	933.0125	4.6	C_20_H_29_N_10_O_23_P_5_	Guanosine pentaphosphate adenosine	1.4	+
(2)	4.87	606.3497	4.3	C_26_H_43_N_11_O_6_	Dynorphin B (6-9)	1.7	+
(3)	4.69	761.3036	1.1	C_39_H_44_N_4_O_12_	Bilirubin glucuronide	1.3	+
(4)	1.97	566.4172	1.4	C_29_H_60_NO_7_P	LysoPE (0:0/24:0)	1.1	+
(5)	2.18	442.0211	9.0	C_10_H_15_N_5_O_11_P_2_	Guanosine diphosphate	1.5	−
(6)	5.27	283.2648	1.8	C_18_H_36_O_2_	Stearic acid	2.3	−
(7)	4.82	423.3265	0.9	C_29_H_44_O_2_	alpha-Tocotrienol	3.5	−
(8)	4.41	327.2318	3.7	C_22_H_32_O_2_	Docosahexaenoic acid	4.6	−
(9)	4.48	373.2375	2.4	C_23_H_34_O_4_	Calcitroic acid	1.0	−
(10)	4.28	482.9574	8.1	C_9_H_15_N_2_O_15_P_3_	Uridine triphosphate	1.4	−
(11)	4.69	301.2370	4.7	C_17_H_34_O_4_	MG (0:0/14:0/0:0)	1.0	−
(12)	5.39	309.2804	1.6	C_20_H_38_O_2_	Eicosenoic acid	1.2	−
